# Daphnetin inhibits proliferation and inflammatory response in human HaCaT keratinocytes and ameliorates imiquimod-induced psoriasis-like skin lesion in mice

**DOI:** 10.1186/s40659-020-00316-0

**Published:** 2020-10-20

**Authors:** Jintao Gao, Fangru Chen, Huanan Fang, Jing Mi, Qi Qi, Mengjuan Yang

**Affiliations:** 1 College of Biotechnology, Guilin Medical University, Guilin, 541100 Guangxi People’s Republic of China; 2 Department of Dermatology, Affiliated Hospital of Guilin Medical University, Guilin, 541001 Guangxi People’s Republic of China

**Keywords:** Daphnetin, Proliferation, Inflammatory response, NF-κb signaling pathway, Psoriasis

## Abstract

**Background:**

Psoriasis is a common chronic inflammatory skin disease. Keratinocytes hyperproliferation and excessive inflammatory response contribute to psoriasis pathogenesis. The agents able to attenuate keratinocytes hyperproliferation and excessive inflammatory response are considered to be potentially useful for psoriasis treatment. Daphnetin exhibits broad bioactivities including anti-proliferation and anti-inflammatory. This study aims to evaluate the anti-psoriatic potential of daphnetin in vitro and in vivo, and explore underlying mechanisms.

**Methods:**

HaCaT keratinocytes was stimulated with the mixture of IL-17A, IL-22, oncostatin M, IL-1α, and TNF-α (M5) to establish psoriatic keratinocyte model in vitro. Cell viability was measured using Cell Counting Kit-8 (CCK-8). Quantitative Real-Time PCR (qRT-PCR) was performed to measure the mRNA levels of hyperproliferative marker gene keratin 6 (KRT6), differentiation marker gene keratin 1 (KRT1) and inflammatory factors IL-1β, IL-6, IL-8, TNF-α, IL-23A and MCP-1. Western blotting was used to detect the protein levels of p65 and p-p65. Indirect immunofluorescence assay (IFA) was carried out to detect p65 nuclear translocation. Imiquimod (IMQ) was used to construct psoriasis-like mouse model. Psoriasis severity (erythema, scaling) was scored based on Psoriasis Area Severity Index (PASI). Hematoxylin and eosin (H&E) staining was performed to examine histological change in skin lesion. The expression of inflammatory factors including IL-6, TNF-α, IL-23A and IL-17A in skin lesion was measured by qRT-PCR.

**Results:**

Daphnetin attenuated M5-induced hyperproliferation in HaCaT keratinocytes. M5 stimulation significantly upregulated mRNA levels of IL-1β, IL-6, IL-8, TNF-α, IL-23A and MCP-1. However, daphnetin treatment partially attenuated the upregulation of those inflammatory cytokines. Daphnetin was found to be able to inhibit p65 phosphorylation and nuclear translocation in HaCaT keratinocytes. In addition, daphnetin significantly ameliorate the severity of skin lesion (erythema, scaling and epidermal thickness, inflammatory cell infiltration) in IMQ-induced psoriasis-like mouse model. Daphnetin treatment attenuated IMQ-induced upregulation of inflammatory cytokines including IL-6, IL-23A and IL-17A in skin lesion of mice.

**Conclusions:**

Daphnetin was able to attenuate proliferation and inflammatory response induced by M5 in HaCaT keratinocytes through suppression of NF-κB signaling pathway. Daphnetin could ameliorate the severity of skin lesion and improve inflammation status in IMQ-induced psoriasis-like mouse model. Daphnetin could be an attractive candidate for future development as an anti-psoriatic agent.

## Background

Psoriasis is a common chronic inflammatory skin disease affecting 2–3% of the population worldwide [1]. It is characterized by excessive proliferation, aberrant differentiation of keratinocytes and inflammatory cells infiltration into the dermis and epidermis [2]. The exact pathogenesis of psoriasis still remains unclear, but it is generally believed that abnormal crosstalk between keratinocytes and immune cells plays important roles in the pathogenesis and development of psoriasis. The stimulation on keratinocytes from various cytokines secreted by immune cells in lesion may contribute to keratinocytes hyperproliferation, and in turn hyperproliferative keratinocytes respond to those cytokines to produce massive proinflammatory cytokines to sustain or even amplify inflammatory response [3–6]. Hence, the strategies able to attenuate keratinocytes hyperproliferation or/and excessive inflammation are considered to be potentially useful for psoriasis treatment.

NF-κB signaling, a crucial pathway to regulate a variety of cellular processes, including proliferation and inflammation [7], was found to be activated in psoriatic lesions and participates in the pathogenesis of psoriasis [8, 9]. Inhibition of proliferation and inflammation in keratinocytes through the inactivation of NF‑κB signaling pathway may represent a novel treatment of psoriasis [8, 10].

Daphnetin (7,8-Dihydroxycoumarin), a natural coumarin compound isolated from *Daphne odora var.*, exhibits broad bioactivities including anti-proliferation and anti-inflammation [11, 12]. Daphnetin was found to inhibit A549 human lung adenocarcinoma cell proliferation by inducing apoptosis via suppression of Akt/NF-κB signaling [13]. Daphnetin ameliorates 7,12-dimethylbenz [a] anthracene-induced mammary carcinogenesis through dual inhibition on Nrf-2-Keap1 and NF-κB pathways [14]. Daphnetin conferred substantial protection from endotoxin-induced acute lung injury through induction of TNFAIP3, thereby inhibiting NF-κB pathways [15]. Daphnetin attenuates acute pancreatic injury in rat by reducing TLR4 expression and inhibiting NF-κB signaling pathway [16]. We have previously shown that daphnetin inhibits inflammation in the systemic lupus erythematosus murine model via inhibition of NF-κB activity [17]. However, the anti-psoriatic potential of daphnetin has not been evaluated.

The cocktail of IL-17A, IL-22, oncostatin M, IL-1α, and TNF-α (M5) cytokines induces keratinocytes manifesting some features of psoriatic keratinocyte in vitro [18]. Therefore, M5 cytokines cocktail was used to establish psoriatic keratinocyte model in vitro [19–21]. Imiquimod (IMQ), an agonist of Toll-like receptor 7/8 ligand, induced a dermatitis in mice closely resembling human psoriasis [22]. Thus, IMQ are widely used to induce psoriasis-like mouse model. In this study, we tried to evaluate the anti-psoriatic potential of daphnetin in vitro and in vivo, and explore underlying mechanisms.

## Results

### The effect of daphnetin on cell viability of HaCaT keratinocytes

**Fig. 1:**
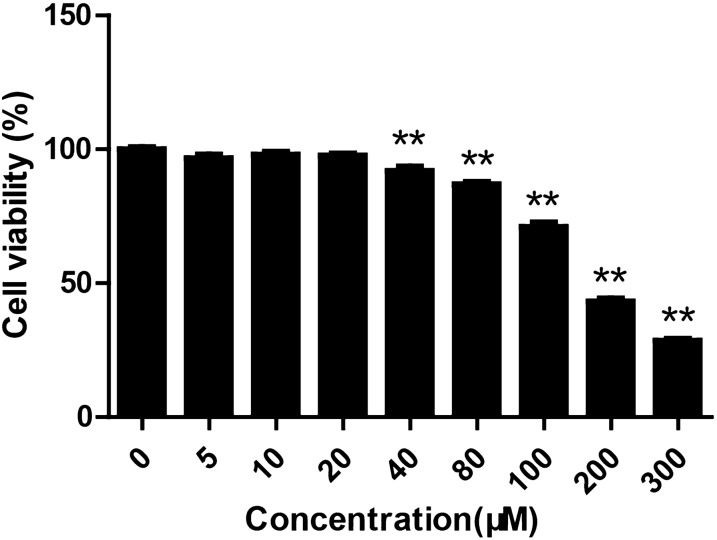
The effect of daphnetin on cell viability of HaCaT keratinocytes. HaCaT keratinocytes were seeded in 96-well plates and treated with different concentrations of daphnetin (0, 5, 10, 20, 40, 35, 80, 100, 200 and 300 μM) for 96 h. CCK-8 was used to measure cell viability. **P < 0.01

The cell viability of HaCaT keratinocytes treated with daphnetin was measured using CCK-8 assay. No significant toxicity was observed at concentrations below 20 μM after treatment with daphnetin for 96 h. Daphnetin at concentrations above 40 μM significantly decreased cell viability of HaCaT keratinocytes (Fig. 1). Therefore, in the current study, daphnetin was used with concentration no more than 20 μM.

### M5 stimulation promoted cell proliferation and inflammatory cytokines expression in HaCaT keratinocytes

**Fig. 2:**
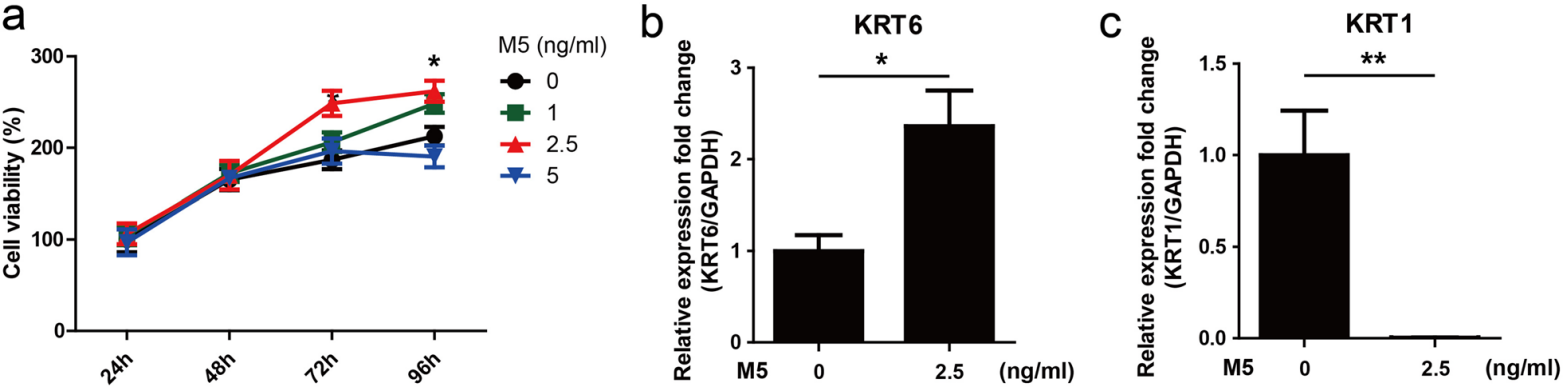
The effect of M5 on the proliferation of HaCaT keratinocytes. **a** HaCaT keratinocytes were seeded in 96-well plates and stimulated with M5 (0, 1, 2.5 and 5 ng/ml) for 24–96 h. CCK-8 was used to measure cell viability. *P < 0.05, M5 (0 ng/ml) group vs M5 (2.5 ng/ml) group. **b**, **c** HaCaT keratinocytes were seeded in 12-well plates and stimulated with M5 (2.5 ng/ml) for 72 h. Cells were harvested and RNA was extracted for qRT-PCR analysis of KRT6 and KRT1 mRNA levels. GAPDH served as an internal reference. *P < 0.05. **P < 0.01

**Fig. 3:**
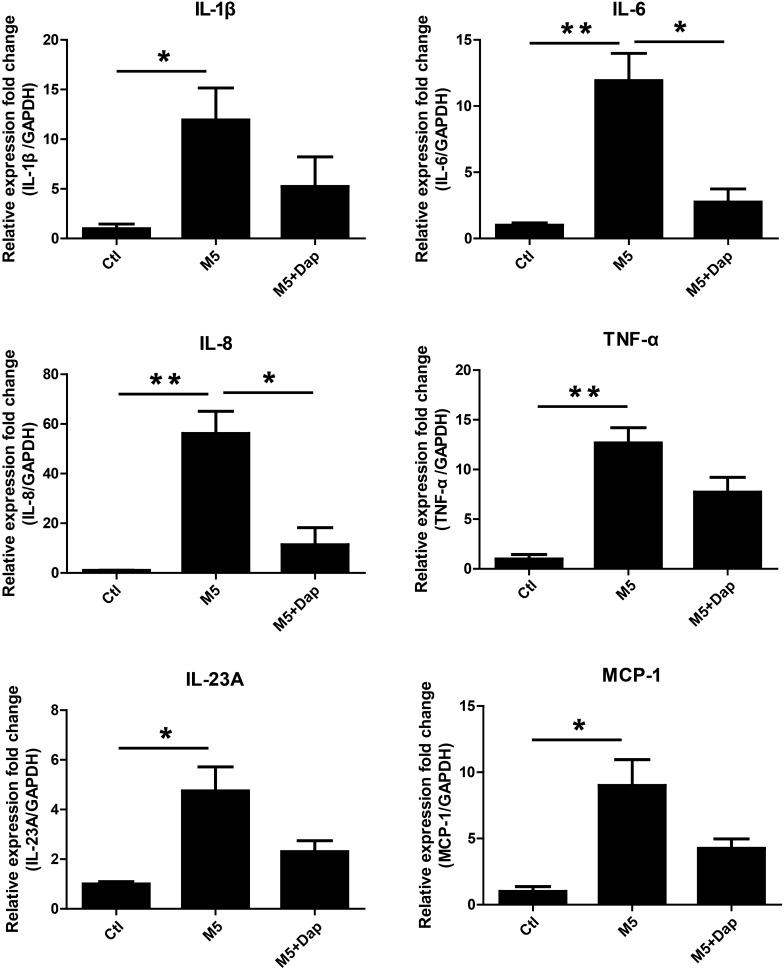
The effect of daphnetin on the expression of inflammatory cytokines in M5-stimulated HaCaT keratinocytes. HaCaT keratinocytes were seeded in 12-well plates and treated with daphnetin (Dap, 20 μM) or not for 2 h, and subsequently stimulated with M5 cytokines (2.5 ng/ml) or not for 24 h. Cells were harvested and RNA was extracted for qRT-PCR analysis of IL-1β, IL-6, IL-8, TNF-α, IL-23A and MCP-1 mRNA levels. GAPDH served as an internal reference. *P < 0.05. **P < 0.01. DMSO (Ctl), DMSO + M5 (M5), daphnetin + M5 (M5 + Dap)

The mixture of IL-17A, IL-22, oncostatin M, IL-1α, and TNF-α (M5) cytokines were used to simulate HaCaT keratinocytes to establish psoriatic keratinocyte model recapitulating some features of psoriasis in vitro [18–20]. M5 (2.5 ng/ml) stimulation for 72 h and 96 h significantly promoted proliferation of HaCaT keratinocytes (Fig. 2a). Keratin 6 (KRT6) was considered as a hallmark of psoriatic keratinocytes hyperproliferation [23, 24]. qRT-PCR result showed that M5 (2.5 ng/ml) stimulation significantly increased KRT6 mRNA level (Fig. 2b). Keratin 1 (KRT1), a keratinocyte differentiation marker [25, 26], was found decreased after treatment with M5 (2.5 ng/ml) (Fig. 2c). In addition, M5 (2.5 ng/ml) stimulation significantly upregulated mRNA levels of IL-1β, IL-6, IL-8, TNF-α, IL-23A and MCP-1 (Fig. 3). These results showed that M5 (2.5 ng/ml) simulation was able to induce HaCaT keratinocytes recapitulating some features of psoriatic keratinocytes. Thus, M5 (2.5 ng/ml) was used to stimulate HaCaT keratinocytes to establish psoriatic keratinocyte model in vitro.

### Daphnetin attenuated M5-induced hyperproliferation and inflammatory cytokines expression in HaCaT keratinocytes

**Fig. 4:**
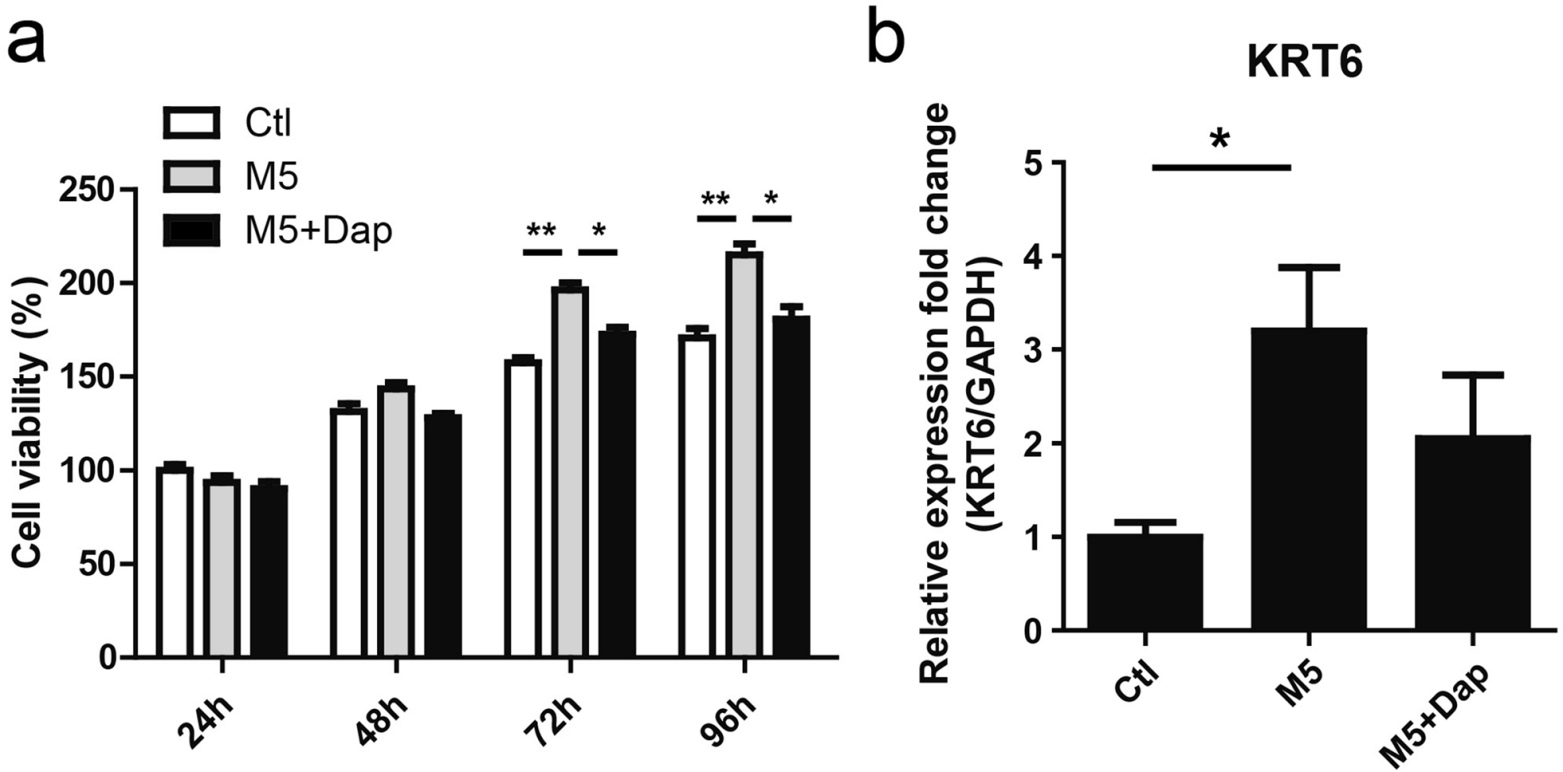
The effect of daphnetin on the proliferation of HaCaT keratinocytes stimulated with M5. **a** HaCaT keratinocytes were seeded in 96-well plates and treated with daphnetin (Dap, 20 μM) or DMSO (Ctl) for 2 h, and subsequently stimulated with M5 cytokines (2.5 ng/ml) or not for 24–96 h. CCK-8 was used to measure cell viability. *P < 0.05. **P < 0.01. **b** HaCaT keratinocytes were seeded in 12-well plates and treated with daphnetin (Dap, 20 μM) or DMSO for 2 h, and subsequently stimulated with M5 cytokines (2.5 ng/ml) or not for 72 h. Cells were harvested and RNA was extracted for qRT-PCR analysis of KRT6 mRNA level. GAPDH served as an internal reference. *P < 0.05. DMSO (Ctl), DMSO + M5 (M5), daphnetin + M5 (M5 + Dap)

In order to evaluate the effects of daphnetin treatment on M5-stimulated HaCaT keratinocytes, CCK8 was used to detect cell viability. The result showed that M5 stimulation for 72 h and 96 h significantly promoted proliferation of HaCaT keratinocytes. However, M5-induced hyperproliferation at 72 h and 96 h was attenuated in the presence of daphnetin (Fig. 4a). M5 stimulation significantly increased KRT6 mRNA level, which was partially reduced in the presence of daphnetin (Fig. 4b). Moreover, compared with M5 treatment alone, some decrease in the expression level of inflammatory cytokines (IL-1β, IL-6, IL-8, TNF-α, IL-23A and MCP-1) was found in HaCaT keratinocytes co-treated with daphnetin and M5. IL-6 and IL-8 were found to be reduced significantly (Fig. 3).

### Daphnetin inhibited p65 phosphorylation and nuclear translocation in HaCaT keratinocytes

**Fig. 5:**
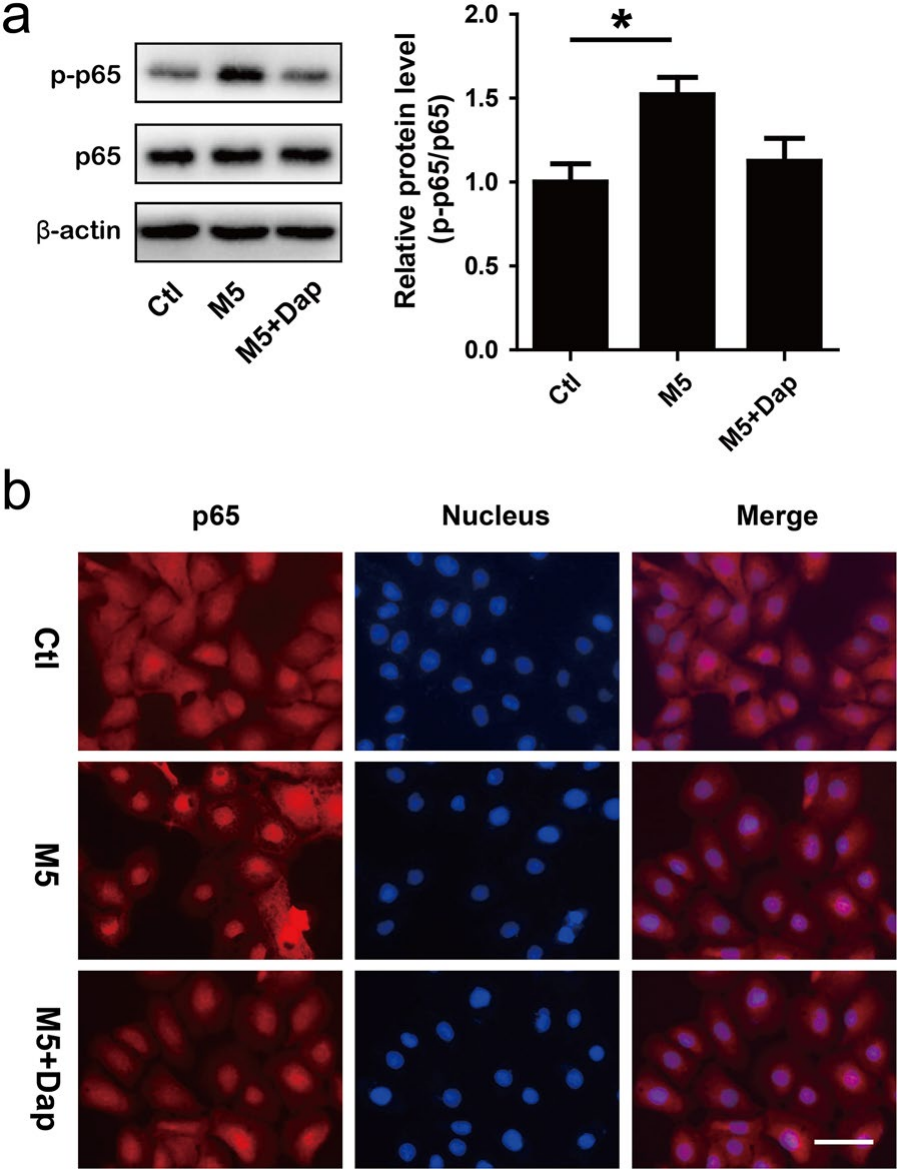
The effects of daphnetin on p65 phosphorylation and nuclear translocation in M5-stimulated HaCaT keratinocytes. HaCaT keratinocytes were treated with daphnetin (Dap, 20 μM) for 24 h, and then stimulated with M5 (5 ng/ml) for 30 min. **a** Cells were harvested and total protein was extracted for Western blotting assay to detect p65 and p-p65 protein levels. β-actin served as an internal reference. *P < 0.05. **b** Cells were fixed and IFA was carried out to detect p65 (red) distribution. Nuclei were stained with Hoechst dye 33,258 (blue). Bar, 50 μm. DMSO (Ctl), DMSO + M5 (M5), daphnetin + M5 (M5 + Dap)

NF-κB signaling pathway plays crucial roles in the pathogenesis of psoriasis [8]. In order to investigate whether daphnetin-mediated inhibition of proliferation and inflammatory response in HaCaT keratinocytes is resulted from NF-κB signaling inactivation, Western blotting and IFA were performed to respectively detect p65 phosphorylation and p65 nuclear translocation. M5 stimulation dramatically induced p65 phosphorylation, which could be restored in the presence of daphnetin (Fig. 5a). p65 resided in cytoplasm in control group (Ctl), and a robust translocation to the nucleus was observed after 30 min stimulation with M5. However, daphnetin treatment retained p65 in cytoplasm (Fig. 5b).

### Daphnetin ameliorated IMQ-induced psoriasis-like skin lesion in mice

**Fig. 6:**
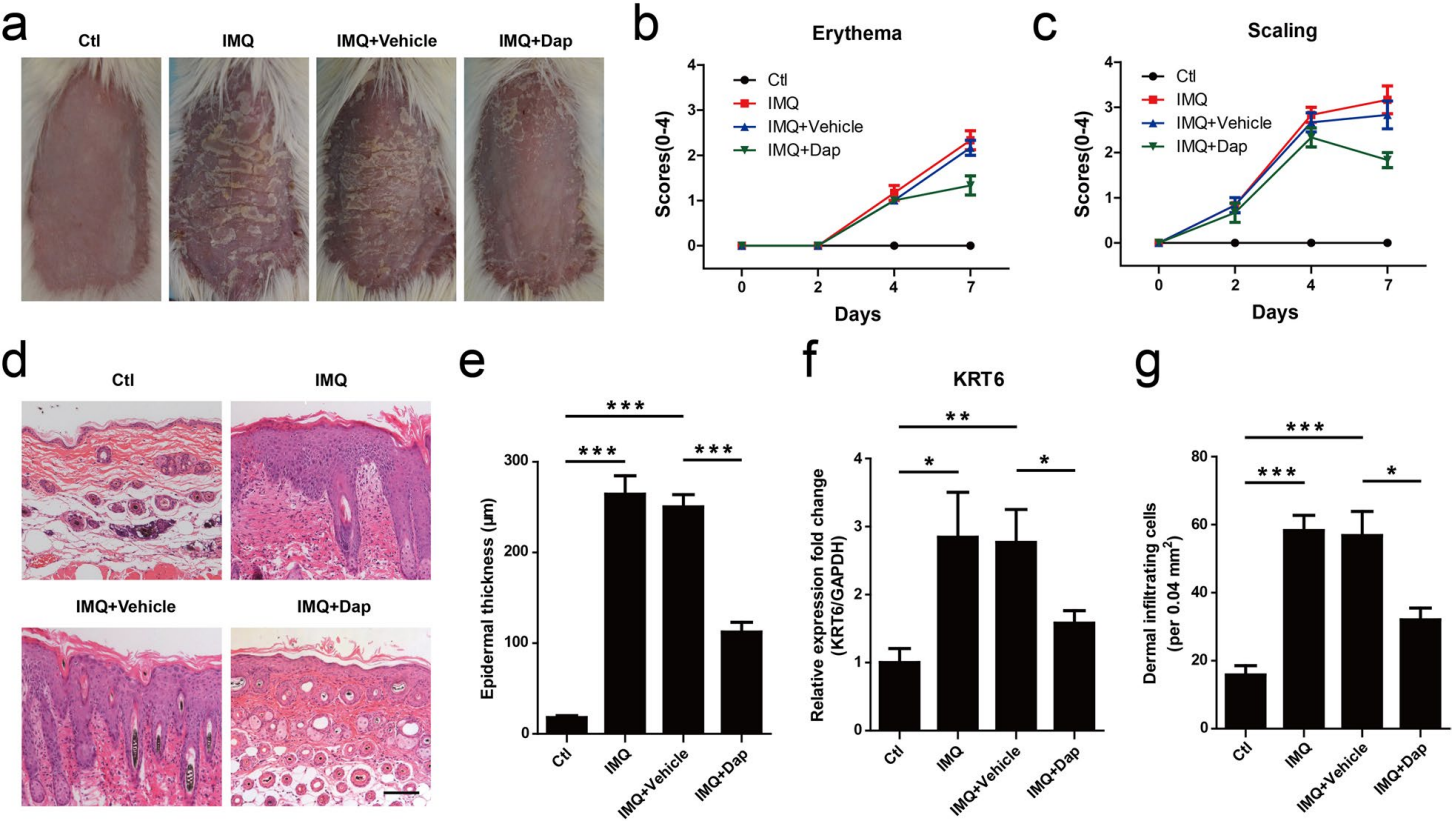
The effect of daphnetin on skin lesion in IMQ-induced psoriasis-like mouse model. **a** The macroscopic appearance of mouse back skin on day 8. **b** Erythema and (**c**) scaling was scored on days 0, 2, 4, and 7 based on the PASI. **d** H&E staining of the mouse skin. Bar, 100 μm. **e** Epidermal thickness was calculated by Image-pro Plus 6.0 software. ***P < 0.001. **f** qRT-PCR was performed to measure the expression of KRT6 in skin lesion. GAPDH served as an internal reference. *P < 0.05. **P < 0.01. **g** Dermal cellular infiltrates were quantitated with H&E staining images. *P < 0.05. ***P < 0.001

To evaluate the effects of daphnetin treatment on IMQ-induced psoriasis-like mouse model, mice was treated with topical daphnetin. The symptoms of erythema and scaling were induced after IMQ treatment. Vehicle cream treatment could not improve those symptoms. However, compared with vehicle group, topical application of daphnetin ameliorated the skin condition (Fig. 6a). The severity of skin lesions (erythema, scaling) was scored on days 0, 2, 4 and 7 based on PASI. Consistent with the Fig. 5a, the mice treated with daphnetin had lower score (erythema, scaling) than vehicle group on day 7 (Fig. 6b, c). Histopathological analysis showed that the mice treated with IMQ had thicker epidermal layers than those of normal group. Vehicle cream treatment could not decrease epidermal thickness. However, epidermal thickness was significantly reduced in the daphnetin group as compared with the vehicle group (Fig. 6d, e). IMQ treatment induced KRT6 expression, which could be reduced with daphnetin treatment (Fig. 6f).

### Daphnetin inhibited inflammatory cytokines expression in IMQ-induced psoriasis-like skin lesion

**Fig. 7:**
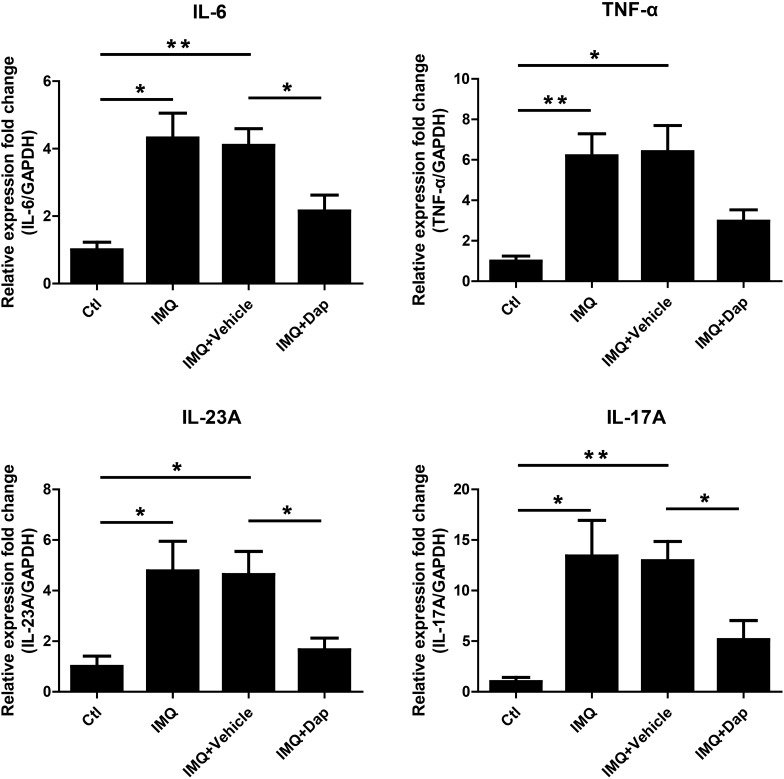
The effect of daphnetin on the expression of inflammatory cytokines in IMQ-induced psoriasis-like skin lesion. qRT-PCR was performed to measure the expression of IL-6, TNF-α, IL-23A and IL-17A in skin lesion. GAPDH served as an internal reference. *P < 0.05. **P < 0.01

Histopathological results showed that daphnetin inhibited inflammatory cell infiltration to the dermis as compared with vehicle cream treatment (Fig. 6d, g), suggesting daphnetin may affect inflammation status in IMQ-induced psoriasis-like skin lesion. qRT-PCR was performed to detect the expression of IL-6, TNF-α, IL-23A and IL-17A. IMQ significantly upregulated mRNA levels of IL-6, TNF-α, IL-23A and IL-17A. However, daphnetin treatment attenuated IMQ-induced upregulation of those inflammatory cytokines in IMQ-induced psoriasis-like skin lesion at a certain extent, significantly reduced the expression of IL-6, IL-23A and IL-17A (Fig. 7).

## Discussion

Psoriasis is a complicated inflammatory skin disease mediated by various kinds of cells, including keratinocytes, T cells, endothelial cells, macrophages and dendritic cells [27]. Keratinocyte, a kind of resident skin cell, is both participant and victim. The balance between proliferation and apoptosis in keratinocytes is crucial for maintaining skin homeostasis. In psoriatic lesions, the balance has been found to be broken. Diminished apoptosis of keratinocytes has been observed, which may resulted in the hyperproliferation of keratinocytes inevitably [28, 29]. Notably, the hyperproliferative keratinocytes express a plethora of cytokines to sustain and amplify the inflammatory response [4–6]. The agents capable of attenuating hyperproliferation or/and massive inflammatory response in keratinocyte could be potential therapeutic drugs for psoriasis.

Daphnetin is a natural coumarin compound isolated from *Daphne odora var.*, with broad pharmacological activities, including anti-proliferation and anti-inflammatory [11, 17, 30]. However, the effects of daphnetin on keratinocytes proliferation and inflammatory response are still not clear. The cocktail of IL-17A, IL-22, oncostatin M, IL-1α, and TNF-α (M5) cytokines were used to simulate keratinocytes to establish psoriatic keratinocyte model in vitro [18–20]. In the current study, M5 (2.5 ng/ml) simulation was found to be able to induce HaCaT keratinocytes manifesting some features of psoriatic keratinocytes (Fig. 2). Interestingly, Daphnetin treatment attenuated the hyperproliferation induced by M5 (Fig. 4a) and partially decreased psoriatic hyperproliferative hallmark KRT6 expression (Fig. 4b).

In order to investigate the effects of daphnetin on the inflammatory response of HaCaT keratinocytes, qRT-PCR was performed to measure the expression of cytokines IL-1β, IL-6, IL-8, TNF-α, IL-23A and MCP-1. IL-1β, IL-6 and TNF-α are important proinflammatory cytokins, especially TNF-α, involving in psoriasis pathogenesis [31, 32]. The expression of IL-1β, IL-6 and TNF-α were elevated with M5 stimulation. Compared with M5 treatment group, IL-1β, IL-6 and TNF-α expression were partially downregulated, significant difference for IL-6, in the presence of daphnetin in M5-stimulated HaCaT keratinocytes (Fig. 3). IL-8 and MCP-1 chemokines contributes to psoriasis pathogenesis through recruiting neutrophils, monocytes and T cells to psoriatic lesion [33–36]. M5 stimulation significantly upregulated IL-8 and MCP-1 expression, especially IL-8 with more than 50-fold increase (Fig. 3). However, M5-induced upregulation of IL-8 was significantly attenuated with daphnetin. IL-23/IL-17 axis plays crucial roles in psoriasis [37–39]. Consistent with above cytokines results, M5-induced IL-23A upregulation could be partially inhibited after treatment with daphnetin (Fig. 3). These results indicated that daphnetin is able to attenuate M5-induced excessive inflammatory response in HaCaT keratinocytes.

NF-κB signaling pathway regulates a variety of cellular processes, including proliferation and inflammation [7]. It has been found that the NF-κB signaling is activated in psoriatic lesions and participates in the pathogenesis of psoriasis [8, 9]. It is thought that the inhibition of NF-κB signaling pathway to reduce hyperproliferation and excessive inflammation in keratinocytes could be promising anti-psoriatic strategy [8, 10]. Several compounds have been found to exhibit attractive therapeutic potential for psoriasis though inhibiting NF-κB signaling pathway [10, 40–43]. Daphnetin was reported to inhibit proliferation and inflammation by regulating the NF-κB signaling pathway [13, 16, 17]. In order to investigate whether daphnetin-mediated inhibition of proliferation and inflammatory response in HaCaT keratinocytes is resulted from NF-κB signaling inactivation, p65 phosphorylation and p65 nuclear translocation were detected. p65 phosphorylation and nuclear translocation are two important events during the activation of NF-κB signaling pathway. In the resting state, combined with IκBα, p65 is sequestered in cytoplasm. Upon stimulation, IκBα is phosphorylated and degraded, resulting in the release of p65 and subsequently translocation into nucleus. In the nucleus, p65 binds to promoters of target genes and drives transcription. During the process of p65 release and translocation, it can be phosphorylated to allow for optimal transcriptional activity [44]. Western blotting analysis indicated that M5 stimulation significantly induced p65 phosphorylation, which could be inhibited in the presence of daphnetin (Fig. 5a). IFA assay showed that M5 stimulation led to robust translocation of p65 into nucleus. However, daphnetin treatment retained p65 in cytoplasm (Fig. 5b). These results indicated that daphnetin could inhibit NF-κB activation.

We further evaluated the effects of daphnetin on IMQ-induced psoriasis-like mouse model. IMQ, an agonist of Toll-like receptor 7/8 ligand, was found to be able to induce a dermatitis in mice closely resembling human psoriasis [22]. Thus, IMQ are widely accepted to induce psoriasis-like mouse model. Consistently, the mice treated with IMQ exhibited some symptoms of psoriasis-like lesions, including erythema, scaling, epidermal hyperplasia and inflammatory cell infiltration. Interestingly, daphnetin treatment significantly ameliorated those symptoms (Fig. 6). qRT-PCR assay showed that daphnetin treatment attenuated IMQ-induced upregulation of inflammatory cytokines including IL-6, IL-23A and IL-17A in skin lesion of mice (Fig. 7). These results indicated that daphnetin could improve IMQ-induced psoriasis-like skin lesion and inflammation status in mice.

## Conclusions

In conclusion, daphnetin was able to attenuate proliferation and inflammatory response induced by M5 cytokines in HaCaT keratinocytes. These biological effects of daphnetin on HaCaT keratinocytes were associated with NF-κB signaling pathway inactivation. Moreover, daphnetin could ameliorate the severity of skin lesion and improve inflammation status in IMQ-induced psoriasis-like mouse model. These results indicated that daphnetin possesses anti-psoriatic potential, which make it an attractive candidate for future developing as an anti-psoriatic agent.

## Methods

### Chemicals and reagents

DMSO (dimethyl sulfoxide) and Hoechst dye 33,258 for nucleus staining were purchased from Sigma-Aldrich (St. Louis, USA). Daphnetin (7,8-Dihydroxycoumarin, CAS 486–35-1) was obtained from PharmaBlock Sciences (Nanjing, China) and dissolved in DMSO. Carbopol 940 and azone were purchased from Rhawn (Shanghai, China). IMQ cream was obtained from Sichuan Med-Shine Pharmaceutical Co., LTD (Sichuan, China). IL-17A, IL-22, oncostatin M, IL-1α, and TNF-α cytokines were purchased from PeproTech (RockyHill, USA). Cell Counting Kit-8 (CCK-8) was purchased from Dojindo (Kumamoto, Japan). TRIzol™ reagent and primers were obtained from Invitrogen (Carlsbad, USA). RevertAid First Strand cDNA Synthesis Kit was purchased from Thermo Fisher Scientific (USA). SYBR Green PCR Master Mix kit was purchased from TaKaRa (Japan). Rabbit anti-p65, anti-p-p65 (Ser536), anti-β-actin primary antibodies and Alexa Fluor 555-labeled anti-rabbit secondary antibody were purchased from Cell Signaling Technology (Beverly, USA).

### Cell culture and exposure to M5 cocktail cytokines

Human HaCaT keratinocytes were obtained from Kunming Cell Bank of Type Culture Collection, Chinese Academy of Science (Kunming, China). Cells were maintained in Dulbecco’s modified Eagle’s medium (DMEM) containing 10% Fetal Bovine Serum (FBS) and cultured in incubator with 5% CO_2_ at 37 °C. Psoriasis-like keratinocytes model was established by adding M5 cocktail cytokins (IL-17A, IL-22, oncostatin M, IL-1α, and TNF-α, each at a final concertration of 2.5 ng/ml) into medium of HaCaT keratinocytes.

### Cell viability assay

CCK-8 was used to measure cell viability. HaCaT keratinocytes were plated in 96-well plates and cultured for 24 h. Cells at 50–60% confluence were treated with daphnetin or M5 cytokines for 24–96 h. 10 μl of CCK-8 solution was added to each well and incubated at 37 °C with 5% CO_2_ for 2 h. Absorbance value at 450 nm was measured using Multiskan Spectrum (Thermo Fisher Scientific, USA) and was directly proportional to the number of living cells.

### Western blotting analysis

HaCaT keratinocytes were seeded in 6-well plates and cultured for 24 h. Cells at 60–80% confluence were treated with daphnetin (20 μM) for 24 h, and then stimulated with M5 (5 ng/ml) for 30 min. Cells was harvested and lysed with cell lysis buffer (Beyotime Biotechnol, China) containing 1 mM phenylmethyl-sulfonylfluoride (PMSF) for 20 min on the ice. Cell lysates were centrifuged for 15 min at 14,000 rpm. The concentration of supernatant protein was determined using BCA kit (Pierce, USA). About 15 μg of protein was separated with sodium dodecyl sulfate–polyacrylamide gel electrophoresis (SDS-PAGE) and transferred to polyvinyl difluoride (PVDF) membranes, which subsequently were blocked using 5% nonfat milk at room temperature for 2 h. Next, the membranes were incubated with anti-p65 (1:1000), anti-p-p65 (1:1000) and anti-β-actin (1:1000) primary antibodies overnight at 4 °C. After being washed with TBS-T for three times, the membranes were incubated with HRP-conjugated secondary antibody at room temperature for 2 h. Finally, the membranes were incubated with chemiluminescence substrate (Pierce, USA) and transferred to ChemiDoc™ XRS + System (Bio-Rad, USA) for visualizing of protein signals. The intensity of protein bands was measured using ImageJ software. β-actin served as an internal reference.

### Indirect immunofluorescence assay (IFA)

HaCaT keratinocytes were plated onto coverslips and treated with daphnetin for 24 h, then stimulated with M5 cytokines for 30 min. Cells were washed with phosphate-buffered saline (PBS) and fixed with 4% paraformaldehyde for 10 min. After being washed with PBS for three times, the cells were permeabilized with PBS containing 0.5% Triton X-100 for 15 min, and subsequently were blocked with PBS containing 1% bovine serum albumin (BSA) at room temperature for 30 min. Next, the coverslips were incubated with rabbit anti-p65 (1:500) primary antibody overnight at 4 °C. After being washed with PBS for three times, the coverslips were incubated with Alexa Fluor 555-labeled anti-rabbit secondary antibody (1:1000) for 2 h. Hoechst dye 33,258 was used to stain nucleus for 4 min. After that, the coverslips were washed with PBS for three times and mounted onto the microscope slides. Immunofluorescence was observed using Nikon Eclipse Ti-U fluorescent microscope.

### Animals and ethical statement

Female specific pathogen-free (SPF) BALB/c mice (6–8 weeks) were purchased from Hunan SJA Laboratory Animal Co., Ltd. (Hunan, China). Mice were housed at constant levels of temperature and humidity with a 12-h light/dark cycle and allowed free access to food and water. All experiment protocols were approved by the Ethical Committees of Guilin Medical University (Guilin, China).

### Daphnetin cream preparation and animal treatment

The composition of daphnetin gel (containing 1% daphnetin) is as follows: daphnetin 0.1 g, carbopol 940 0.2 g, azone 0.1 g, ethanol (96%) 3 g and distilled water q.s. to 10 g [45, 46]. The carbopol 940 powder was added to appropriate quantity of distilled water while being stirred. The solution was incubated overnight at room temperature. Daphnetin was dispersed in ethanol (96%) and added with azone. The daphnetin solution was transferred to the aqueous solution of carbopol 940 while being stirred continuously until the gel formed. The vehicle cream was also prepared without daphnetin.

**Fig. 8:**
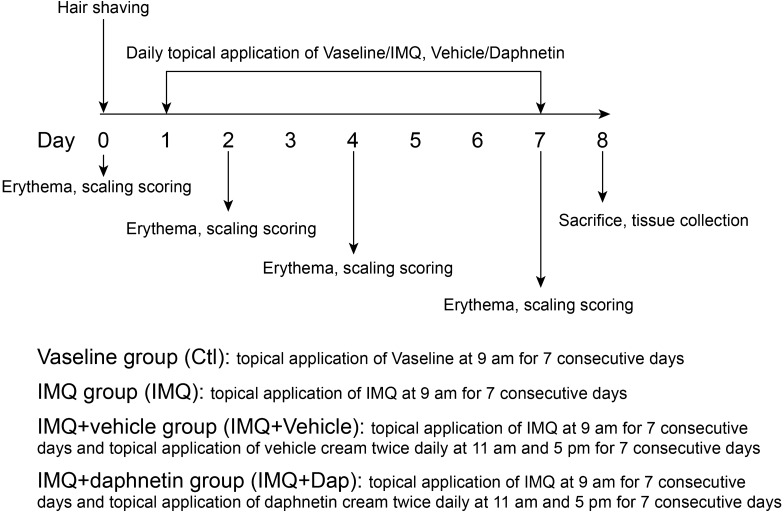
The diagram depicting the experimental design for animal treatment

Mice were randomly divided into four groups (n = 6 per group): Vaseline group (Ctl), IMQ group (IMQ), IMQ + vehicle group (IMQ+Vehicle) and IMQ + daphnetin group (IMQ+Dap). A daily topical dose of 62.5 mg of IMQ cream or vaseline was used on the back skin of the mice for 7 consecutive days. A dose of 50 mg/cm^2^ daphnetin cream or vehicle cream (without daphnetin) was applied twice daily for 7 consecutive days [46]. Erythema and scaling were scored on days 0, 2, 4 and 7 from 0 to 4 based on the Psoriasis Area Severity Index (PASI): 0, none; 1, 2, moderate; 3, severe; 4, very severe. On day 8, the mice were euthanized and skin samples were collected for Hematoxylin and eosin (H&E) staining and RNA extraction. The diagram depicting the experimental design is shown in Fig. 8.

### Histological analysis

Skin tissues of mice were fixed in 4% paraformaldehyde solution and embedded in paraffin. The paraffin-embedded tissues were sectioned and stained with hematoxylin and eosin (H&E) for histological evaluation. Epidermal thickness was calculated by Image-pro Plus 6.0 software. The number of infiltrating cells in dermis was calculated with 5 randomly selected areas coverage of 0.04 mm^2^ per sample.

### RNA extraction and quantitative real-time PCR analysis

HaCaT keratinocytes were seeded in 12-well plates and treated with daphnetin (Dap, 20 μM) or not for 2 h, and subsequently stimulated with M5 cytokines (2.5 ng/ml) or not for 24/72 h. Cells were harvested and RNA was extracted for qRT-PCR analysis of KRT6, IL-1β, IL-6, IL-8, TNF-α, IL-23A and MCP-1 mRNA levels. As shown in Fig. 8, the mice were euthanized and skin samples were collected on day 8. The RNA was extracted for qRT-PCR analysis of IL-6, TNF-α, IL-23A and IL-17A.

Total RNA was extracted from HaCaT keratinocytes or mice skin tissue using TRIzol™ reagent according to the manufacturer’s instruction. First strand of cDNA was synthesized with 2 μg of total RNA using RevertAid First Strand cDNA Synthesis Kit. Quantitative Real-Time PCR (qRT-PCR) was performed with SYBR Green PCR Master Mix kit in CFX96 Touch™ Real-Time PCR Detection System (Bio-Rad, USA). The qRT-PCR reaction procedure was 10 s for denaturation at 95 °C, 10 s for annealing at 60 °C and 10 s for extension at 72 °C, with 40 cycles in total. GAPDH served as internal reference. The relative expression was analyzed by the 2^−ΔΔCt^ method [47]. Primers sequences targeting genes in HaCaT keratinocytes were as follows:

- KRT6, 5′-GGGTTTCAGTGCCAACTCAG-3′ (forward) and 5′-CCAGGCCATACAGACTGCGG-3′ (reverse), 146 bp (product size);
- KRT1, 5′-CTTTTCTGCTGTTTCCCAATGAA-3′ (forward) and 5′-GGAAAGAACAAAGCAGGGTCATAG-3′ (reverse), 80 bp (product size);
- IL-1β, 5′-ATGATGGCTTATTACAGTGGCAA-3′ (forward) and 5′-GTCGGAGATTCGTAGCTGGA-3′ (reverse), 132 bp (product size);
- IL-6, 5′-ACTCACCTCTTCAGAACGAATTG-3′ (forward) and 5′-CCATCTTTGGAAGGTTCAGGTTG-3′ (reverse), 149 bp (product size);
- IL-8, 5′-ACTGAGAGTGATTGAGAGTGGAC-3′ (forward) and 5′-AACCCTCTGCACCCAGTTTTC-3′ (reverse), 112 bp (product size);
- TNF-α, 5′-CCTCTCTCTAATCAGCCCTCTG-3′ (forward) and 5′-GAGGACCTGGGAGTAGATGAG-3′ (reverse), 220 bp (product size);
- IL-23A, 5′-CTCAGGGACAACAGTCAGTTC-3′ (forward) and 5′-ACAGGGCTATCAGGGAGCA-3′ (reverse), 119 bp (product size);
- MCP-1, 5′-CAGCCAGATGCAATCAATGCC-3′ (forward) and 5′-TGGAATCCTGAACCCACTTCT-3′ (reverse), 190 bp (product size);
- GAPDH, 5′-CACATGGCCTCCAAGGAGTAA-3′ (forward) and 5′-TGAGGGTCTCTCTCTTCCTCTTGT-3′ (reverse), 75 bp (product size).

Primers sequences targeting genes in mice were as follows:

- KRT6, 5′-CTGTGAGTTTCTAATGGCCTGAGA-3′ (forward) and 5′-GAAACTTACATCACAGGACCAGTGA-3′ (reverse), 102 bp (product size);
- IL-6, 5′-CCTCTCTGCAAGAGACTTCCAT-3′ (forward) and 5′-AGTCTCCTCTCCGGACTTGT-3′ (reverse), 95 bp (product size);
- TNF-α, 5′-ATCCGCGACGTGGAACTG-3′ (forward) and 5′-ACCGCCTGGAGTTCTGGAA-3′ (reverse), 70 bp (product size);
- IL-23A, 5′-TCCTCCAGCCAGAGGATCACCC-3′ (forward) and 5′-AGAGTTGCTGCTCCGTGGGC-3′ (reverse), 160 bp (product size);
- IL-17A, 5′-CCTCACACGAGGCACAAGTG-3′ (forward) and 5′-CTCTCCCTGGACTCATGTTTGC-3′ (reverse), 68 bp (product size);
- GAPDH, 5′-AGCTTGTCATCAACGGGAAG-3′ (forward) and 5′-TTTGATGTTAGTGGGGTCTCG-3′ (reverse), 62 bp (product size).

## Statistical analysis

Data were presented as mean ± standard error of mean (SEM) from at least three independent experiments. GraphPad Prism software was used for statistical analysis. Statistical significance analysis was performed using Student’s *t* test. *P* value < 0.05 was considered statistically significant.

## Data Availability

All data generated or analyzed during this study are included in this published article.

## Abbreviations

Dap: Daphnetin
DMSO: Dimethyl sulfoxide
CCK-8: Cell counting kit-8
SDS-PAGE: Sodium dodecyl sulfate–polyacrylamide gel electrophoresis
PVDF: Polyvinyl difluoride
PBS: Phosphate-buffered saline
TBS-T: Tris-buffered saline containing 0.5% Tween-20
PMSF: Phenylmethyl-sulfonylfluoride
IFA: Indirect immunofluorescence assay
IMQ: Imiquimod
H&E staining: Hematoxylin and eosin staining
